# Respiratory and Cardiac Phase Coupling With Voluntary Actions Across Motor Tasks

**DOI:** 10.1111/psyp.70264

**Published:** 2026-02-18

**Authors:** Hiroshi Shibata, Hideki Ohira

**Affiliations:** ^1^ Department of Informatics Nagoya University Nagoya Aichi Japan

**Keywords:** heartbeat, interoception, respiration, sensorimotor synchronization, voluntary action

## Abstract

Bodily rhythms such as breathing and heartbeat influence perception and motor processes. Recent studies have indicated that breathing phases, particularly exhalation, synchronize with voluntary actions, potentially reflecting a general influence on motor intention. However, this effect might depend on the specific effector and movement direction. This study aimed to investigate (i) respiratory synchronization across different voluntary motor tasks, (ii) the interaction between stimulus‐locked and action‐locked respiratory coupling, and (iii) cardiac synchronization with voluntary actions. A total of 32 healthy participants performed two voluntary motor tasks: a modified Libet clock task and an elbow flexion–extension task. In the Libet clock task, the participants monitored a rotating dot on a clock face and either pressed a key at a self‐chosen time (key‐press condition) or released the key after holding it pressed (key‐release condition). In the elbow flexion–extension task, the participants spontaneously pushed (elbow extension) or pulled (elbow flexion) a joystick. Across tasks, voluntary actions showed an overall tendency to occur during exhalation across multiple effectors (finger and elbow) and movement directions (extension and flexion). Furthermore, stimulus‐locked respiratory phase was associated with subsequent action timing, suggesting that trial structure can shape respiration–action coupling. We found no robust evidence for systematic cardiac‐phase modulation of voluntary action timing, although a weak condition‐specific trend was observed. Collectively, these findings support respiration–action coupling across diverse actions and highlight a potential contribution of stimulus‐locked respiratory dynamics to voluntary action timing.

## Impact

Recent evidence suggests voluntary actions synchronize with exhalation, but generalization across diverse motor tasks was unclear. This study demonstrates robust exhalation synchronization for voluntary finger and elbow movements, supporting an exhalation‐driven intention mechanism rather than motor‐specific alignment. Additionally, our findings extend previous research by revealing trial‐by‐trial stimulus‐action respiratory coupling and weak cardiac synchronization effects.

## 1 Introduction

Breathing is a bodily rhythm that can influence perception and behavior. In recent years, a growing body of research has demonstrated that interoception—our representation of bodily states derived from physiological signals (Chen et al. 2021)—influences various aspects of our lives, including emotion and cognition (Critchley and Garfinkel 2017, 2018). Although early studies primarily focused on the cardiac domain (Garfinkel et al. 2014; Tsakiris et al. 2011), accumulating evidence indicates that the respiratory phases, particularly inhalation and exhalation, can modulate perception, cognition, and emotional processing (Mizuhara and Nittono 2023; Perl et al. 2019; Zelano et al. 2016). For instance, an intracranial EEG study has provided neurophysiological evidence for such respiratory influences, suggesting that nasal inhalation is associated with enhanced cortical oscillations and improved performance in certain sensory and memory tasks (Zelano et al. 2016).

Besides modulating sensory processes, respiratory phases also influence motor behavior. Behavioral studies have shown that participants' response timing in cognitive tasks tends to align with specific breathing phases (Harting et al. 2025; Johannknecht and Kayser 2022). Similar respiratory‐motor synchronization has been reported across a wide range of motor behaviors, including eye movements during sleep and wakefulness (Rassler and Raabe 2003; Rittweger and Pöpel 1998), visually guided arm movements (Krupnik et al. 2015), and rhythmic actions such as walking and tapping (Bechbache and Duffin 1977; Ebert et al. 2002; Wilke et al. 1975). Collectively, these findings indicate coupling between respiratory rhythms and various motor processes.

Voluntary action initiation has been reported to show a bias toward exhalation. Park et al. (2020) demonstrated that self‐initiated key presses more frequently occur during late exhalation. In that study, voluntary actions were examined using two tasks: the Libet clock task (Libet et al. 1983), in which a dot rotates on a clock face and participants press a key at a self‐chosen moment, and the Kornhuber task (Kornhuber and Deecke 1965), in which participants continuously perform self‐paced key presses while isolated from external sensory stimuli. Crucially, respiratory phase covaried with readiness potential (RP) amplitudes, indicating a temporal association between respiration and action preparation. Subsequent research extended these findings to cognitive tasks, such as motor and visual imagery, showing similar respiratory biases and RP coupling patterns (Park et al. 2022).

However, previous work has largely examined a single distal effector (finger flexion), leaving open whether exhalation‐aligned timing generalizes across effectors and movement directions. Biomechanical studies indicate that respiratory influences vary with movement characteristics. For example, finger flexor force can peak during forced exhalation (Li and Laskin 2006), elbow extension (but not elbow flexion) can be facilitated during forced exhalation (Ikeda et al. 2009), and respiratory phase can differentially affect the precision of flexion and extension movements (Rassler 2000). These findings raise the possibility that respiration–action coupling may differ across effectors and movement directions. Thus, it remains unclear whether the exhalation‐linked preference established for key presses (Park et al. 2020) also holds for proximal limb actions (elbow flexion and extension) and for actions with opposite kinematics (e.g., finger extension).

Moreover, two critical issues remain unresolved. First, breathing can become synchronized (entrained) to repeated stimulus presentations during cognitive tasks (Goheen et al. 2024; Harting et al. 2025; Johannknecht and Kayser 2022). However, the potential influence of this stimulus‐locked respiratory synchronization on voluntary action timing has not been systematically examined. In the Libet clock task, each trial begins with the presentation of a rotating dot, and these trial onsets are separated by inter‐trial intervals drawn from a restricted temporal range. This temporal structure may entrain respiration to trial onsets, potentially interacting with action‐related respiration coupling and complicating interpretation. Here, we examine both stimulus‐locked and action‐locked respiratory synchronization and explore their characteristics and interrelationship.

Second, the evidence for cardiac synchronization in voluntary actions remains inconsistent. While Park et al. (2020) found no systematic cardiac phase bias during voluntary key presses, Mussini et al. (2024) showed that participants were less likely to initiate actions during systole, supporting the baroreceptor‐mediated inhibitory theories (Makowski et al. 2020; Rae et al. 2018). In contrast, other studies reported increased voluntary action frequency during systole (Kunzendorf et al. 2019) or a tendency to perform actions away from the R‐peak (Palser et al. 2021). These mixed findings may stem from methodological or task‐based differences.

Therefore, the present study investigates (i) whether voluntary actions involving distinct effectors (finger flexion, finger extension, elbow flexion, and elbow extension) exhibit a consistent exhalation‐phase bias; (ii) how respiratory synchronization to stimulus presentations relates to respiratory coupling during voluntary action initiation; and (iii) whether voluntary actions synchronize with cardiac cycles, given the inconsistent evidence currently available. Additionally, we explore whether individual differences in respiratory and cardiac physiology account for variability in respiration–action coupling. Specifically, we examine mean breathing interval and its variability, as well as mean RR interval and heart rate variability (HRV). These indices reflect autonomic tone and respiratory flexibility and may provide mechanistic insights into individual variability in phase‐locking. By addressing these questions, we aim to clarify inconsistencies in the literature and advance understanding of how respiration shapes the temporal organization of voluntary action.

## 2 Methods

### 2.1 Participants

The participants were 32 healthy Japanese students (17 female; 27 right‐handed; mean age: 21.87 ± 2.42 years) attending Nagoya University in Japan. The sample size was determined based on an a priori power analysis. Although the closest paradigm (Park et al. 2020) demonstrated robust respiration–action coupling with *N* ranging from 20 to 32, it did not directly report standardized effect sizes due to the use of circular statistics. Accordingly, for a priori planning, we used a medium effect size (*d* = 0.5) as a conservative proxy for the within‐participant contrast. Assuming a one‐tailed test (*α* = 0.05, power = 0.80), the analysis indicated that a minimum of 27 participants was required to detect a statistically significant effect. We recruited 32 participants to account for potential dropouts. All hypothesis tests reported in the manuscript are two‐tailed unless stated otherwise. In the Libet clock task, data from four participants were excluded because at least one condition contained fewer than 20 valid trials after applying predefined behavioral criteria (see Section 2.5.1), resulting in a final sample size of 28. These exclusions applied only to the Libet clock task analyses; all 32 participants were retained for the elbow task analyses. One participant's ECG data were unusable due to recording issues and were excluded from cardiac‐phase analyses. The Ethics Committee of the Department of Psychology at Nagoya University approved this study (No. NUPSY‐220729‐G‐01). All participants provided written informed consent before participating in the study and were compensated for their participation.

### 2.2 Physiological Recording

Continuous respiration data were recorded using an MP160 acquisition system with an RSP100C respiratory amplifier and SKT100C thermal airflow amplifier (Biopac Systems Inc.) at a sampling rate of 200 Hz. Respiratory movements were measured using a respiration belt transducer (TSD201; Biopac Systems Inc.) placed around each participant's abdomen. Simultaneously, nasal airflow was monitored using a thermal transducer (TSD202A; Biopac Systems Inc.) positioned just below the right nostril and secured within a mask to ensure accurate detection of the airflow temperature changes associated with breathing. Electrocardiogram (ECG) data were recorded using an MP160 system with an ECG100C amplifier (Biopac Systems Inc.) at a sampling rate of 200 Hz. Disposable ECG electrodes were placed below each clavicle (right and left) and on the lower left side of the abdomen. All physiological data were recorded using AcqKnowledge‐NDT software (Biopac Systems Inc.). The participants were instructed to maintain natural nasal breathing throughout the experiment and were not informed that respiratory or cardiac coupling with voluntary actions was of interest.

### 2.3 Tasks

To comprehensively examine whether the respiration–action coupling generalizes across different effectors, we implemented two distinct motor tasks: the Libet clock task (key press via finger flexion, or key release via finger extension) and elbow flexion–extension task (joystick push or pull involving elbow flexion and extension).

#### 2.3.1 Libet Clock Task

**FIGURE 1:**
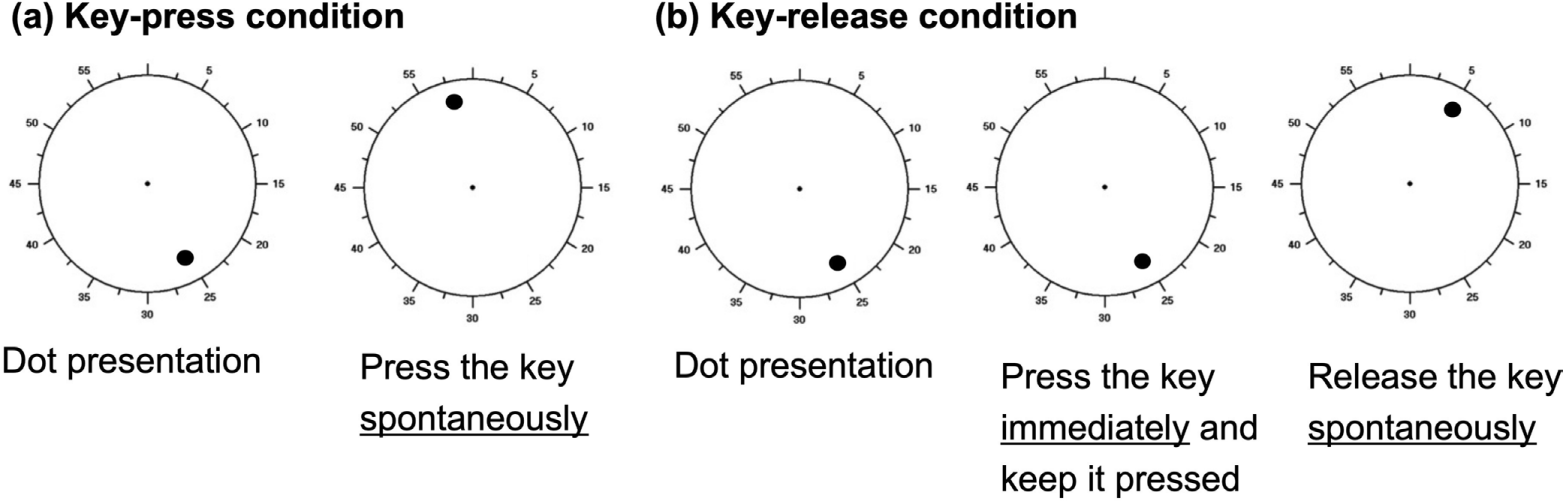
Libet clock task (key‐press and key‐release conditions). The participants viewed a rotating dot (cycle duration: 2560 ms) on the clock face. (a) In the key‐press condition, they spontaneously pressed a key at their chosen moment after at least one full rotation. (b) In the key‐release condition, the participants initially pressed and held a key, spontaneously releasing it after at least one full rotation. Following each action, the rotating dot disappeared. After a random interval of 4–8 s, the dot reappeared, marking the start of the next trial.

Each trial commenced when a black dot appeared at a random position on the clock face (radius: 2° visual angle), rotating at a rate of 2560 ms per cycle (Figure 1). For the key‐press condition, the procedures aligned with those described by Park et al. (2020). The participants were instructed to allow the dot to complete at least one full rotation before freely pressing the key at their chosen moment using their right index finger. Immediately after the key press, the rotating dot disappeared. Each subsequent trial began after a randomized inter‐trial interval of 4–8 s. In the key‐release condition, the participants initially pressed and held the button as soon as the rotating dot appeared. They were instructed to allow the dot to complete at least one full rotation before releasing the key at their chosen moment. Similar to the key‐press condition, the rotating dot disappeared immediately upon key release, and the following trial commenced after a randomized inter‐trial interval of 4–8 s. Consistent with prior research (Libet et al. 1983; Park et al. 2020), the participants were explicitly instructed to avoid preselecting the dot's location for pressing or releasing the key, refrain from maintaining identical intervals between trials, and respond spontaneously. The key‐press and key‐release conditions were applied in a block design in a counterbalanced order. Each condition comprised 40 experimental trials, preceded by 5 practice trials. The participants were offered rest periods after every 20 trials. The timings of dot appearance, key press, and key release were recorded and transmitted to the AcqKnowledge‐NDT software as trigger signals.

#### 2.3.2 Elbow Flexion–Extension Task

**FIGURE 2:**
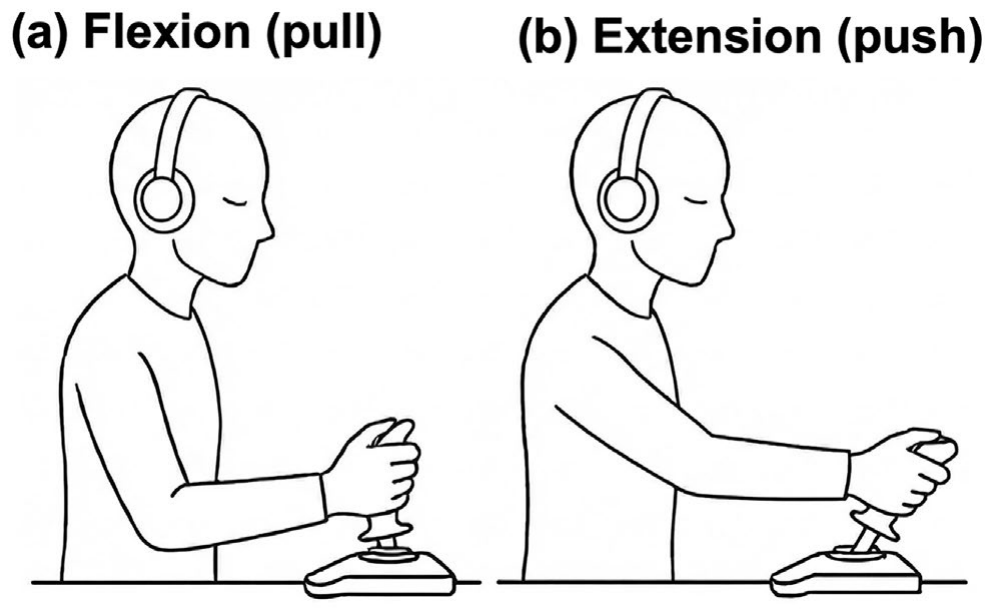
Elbow flexion–extension task (pull and push conditions). The participants operated a joystick positioned in front of the right abdomen. In the pull condition, they spontaneously pulled the joystick toward the body via right‐elbow flexion; in the push condition, they spontaneously pushed it away via elbow extension. Apart from movement direction, the procedure followed the Kornhuber task, with one voluntary joystick movement approximately every 8–12 s. Participants kept their eyes closed and listened to continuous white noise.

The experimental session consisted of two 8‐min blocks (pull and push), counterbalanced across participants. In the pull condition, the participants voluntarily pulled a joystick (Extreme 3D Pro, Logitech) with the right arm via elbow flexion; in the push condition, they voluntarily pushed the joystick with the right arm via elbow extension (Figure 2). Apart from effector direction, the procedure followed the Kornhuber task (Kornhuber and Deecke 1965; Park et al. 2020). The participants were instructed to perform one voluntary joystick movement approximately every 8–12 s. They were explicitly asked to avoid counting seconds, prevent rhythmic or regular joystick movements, and rely solely on spontaneous timing. No mention of respiratory cycles was provided during instruction or practice. The 8–12‐s range was adopted from Park et al. (2020) and was chosen to prevent rapid successive movements while preserving spontaneity and sufficient inter‐event spacing for physiological analyses.

The participants practiced pushing and pulling the joystick using elbow flexion and extension before each condition. Prior to the main recording, they completed a brief training session lasting 1 min to familiarize themselves with the non‐rhythmic, self‐initiated timing requirement. After practice, the intervals between their practice movements were displayed on a monitor. If a participant's intervals deviated markedly from the 8 to 12‐s range, the experimenter asked them to “space movements a little more/a little less”; the main task then proceeded without an additional practice block. Throughout the experiment, the participants were acoustically isolated with continuous white noise delivered via headphones and were instructed to keep their eyes closed. This followed prior work (Park et al. 2020) and was intended to minimize exogenous sensory input and support endogenous, self‐initiated timing.

Joystick position along the y‐axis (normalized range −1 to +1) was sampled at 60 Hz. Movement onset was defined as the first sample at which the signed position exceeded +0.40 in the push block (or fell below −0.40 in the pull block). A 4‐s refractory window suppressed additional detections within a short interval. Onset times were transmitted to AcqKnowledge‐NDT via a trigger signal.

### 2.4 Procedure

The participants were seated comfortably in front of a computer monitor (EV2495‐BK, EIZO), positioned 57 cm away. Stimulus presentation and response collection were controlled using PsychoPy (version 2022.10). After setting up the physiological recording equipment (respiration belt, thermal transducer, and ECG electrodes), the participants completed the Libet clock task (key press or release conditions), followed by the elbow flexion–extension task (joystick push or pull conditions). After completing the tasks, they received a debriefing regarding the study's aims and procedures and answered post‐experiment questionnaires assessing task experience and compliance with instructions. The entire experimental session lasted approximately 90 min.

### 2.5 Data Analysis

#### 2.5.1 Exclusion Criteria for Behavioral Data

In the Libet clock task, trials were excluded when participants in the key‐press condition responded before the rotating dot completed one full rotation (2560 ms). In the key‐release condition, trials were excluded when the initial key press occurred more than 1 s after dot onset or when the participants released the key before completion of the one rotation (2560 ms). Participants who had fewer than 20 valid trials per condition were excluded from subsequent analyses. In the elbow flexion–extension task, trials with inter‐movement intervals shorter than 5 s or longer than 30 s were excluded from the analysis.

Applying these criteria resulted in the exclusion of 7.7% of Libet trials. Four participants were removed from the Libet analyses because they had fewer than 20 valid trials in at least one condition. For the elbow task, 6.3% of trials were excluded, and no participants were removed (see Results).

#### 2.5.2 Pre‐Processing for Physiological Data

Respiration data were band‐pass filtered between 0.1 Hz (high‐pass) and 3 Hz (low‐pass) to cover the physiological respiratory range and suppress slow drift and high‐frequency artifacts (Lorig 2017). Because of the temporal delay introduced by the thermal airflow measurement, the airflow data were corrected to align with the respiration belt data. Specifically, the thermal airflow signal was inverted in phase, and a cross‐correlation analysis with the respiration belt data was conducted to identify the optimal temporal shift that yielded the maximum correlation (Figure S1). All subsequent analyses were performed using the corrected airflow data. To determine instantaneous respiratory phases, we applied the Hilbert transform to the corrected airflow data and obtained the phase angle (0–2π). We defined troughs as phase 0 (and 2π) and peaks as phase π, and classified inhalation as phase 0–π (trough‐to‐peak) and exhalation as phase π–2π (peak‐to‐trough) (Figure S2). Respiratory cycles with abnormal durations were excluded based on participant‐specific criteria, specifically cycles with total cycle, inhalation, or exhalation durations outside the range defined by Q1–2.5 × interquartile range (IQR) and Q3 + 2.5 × IQR. Finally, the respiratory phases at the time of the behavioral events (dot onset, key press or release, and joystick push or pull) were extracted for further analyses. Sensitivity analyses excluding respiratory pauses or omitting the outlier rejection yielded qualitatively identical results (see Figures S8–S11).

We band‐pass filtered ECG signals between 0.5 Hz (high‐pass) and 40 Hz (low‐pass) (Kligfield et al. 2007). R‐peak detection was performed, and instantaneous cardiac phases were calculated based on the detected R‐peaks, consistent with the procedure described by Park et al. (2020) (Figure S3). Abnormal R‐R intervals, which were defined as intervals falling outside the range from Q1–2.0 × IQR to Q3 + 2.0 × IQR, were identified and excluded for each participant. Subsequently, the cardiac phases at the time of the behavioral events were extracted for further analyses. The physiological data were preprocessed using MATLAB (R2024a).

#### 2.5.3 Statistical Analysis

##### 2.5.3.1 Circular Analysis

We investigated the synchronization between voluntary movements and physiological signals (respiratory and cardiac phases) using the methods established by Park et al. (2020). The Hodges–Ajne (omnibus) test was used to determine whether the phase distribution of voluntary movements deviated significantly from uniformity (Ajne 1968). Given that exhalation typically lasts longer than inhalation, random events tend to cluster during exhalation. To control for this bias, we generated 1000 surrogate datasets by randomly shifting the respiratory phase data for each participant, allowing the empirical determination of chance‐level distributions. The observed statistical value (*M*), defined as the minimum number of data points falling within any half‐circle, was compared with these surrogate distributions. Lower *M* values indicated greater deviation from uniformity, and significant departures from the null hypothesis (uniform distribution) were evaluated using two‐tailed permutation tests. All *p*‐values were corrected for multiple comparisons using the false discovery rate (FDR) method. Circular statistical analyses were performed using MATLAB (R2024a) and the Circular Statistics Toolbox (Berens 2009).

Differences in the mean phase across conditions were analyzed following the procedures described by Grund et al. (2022). We applied a randomized version of Moore's paired circular test (Moore 1980) to evaluate the mean phase differences between the conditions. This involved 10,000 random permutations for each comparison. To account for multiple comparisons, we corrected all resulting *p*‐values using the FDR procedure.

##### 2.5.3.2 Physiological State Analysis

Analyses were conducted by dividing the data according to specific physiological states. For the respiration‐based analyses, the phases were classified into inhalation (first half of the respiratory cycle) and exhalation (second half of the respiratory cycle). Cardiac state analyses were conducted by defining a physiologically motivated systolic window, from 200 to 400 ms after each R‐peak (Azevedo et al. 2017), and computing the proportion of events occurring within this window relative to the entire R–R interval (“systole ratio”). For each respiratory and cardiac state, the frequency of events was calculated and expressed as proportions. Because the inhalation ratio is directly complementary to the exhalation ratio (i.e., inhalation ratio = 1—exhalation ratio) and the non‐systole ratio is complementary to the systole ratio, we used only the exhalation and systole ratios in subsequent analyses. The baseline proportions were defined as the temporal proportion of each physiological state (e.g., exhalation, systole) within each condition. An analysis of variance (ANOVA) was conducted with condition and timing as independent variables and exhalation and systole ratios as dependent variables. Subsequent individual *t*‐tests comparing each condition to its baseline were performed, with *p*‐values corrected for multiple comparisons using the FDR method. For the cardiac analysis, Bayes factors were additionally computed using the BayesFactor package in R for specific contrasts to quantify the evidence for the null versus alternative hypotheses.

As a robustness check, we additionally fitted trial‐wise generalized linear mixed‐effects models (GLMMs) predicting whether an action occurred during exhalation (0 = inhalation, 1 = exhalation). For the Libet clock task, fixed effects included condition (key‐press vs. key‐release), respiratory state at dot presentation, cardiac state at action timing, and action interval (i.e., the latency between dot presentation and the voluntary action). For the elbow flexion–extension task, fixed effects included movement condition (pull vs. push) and action interval, defined as the time since the previous action. All models included random intercepts for participants, with each participant's baseline exhalation ratio (logit‐transformed) entered as an offset term. Full model specifications and results are reported in Supplementary Mixed‐effects Analyses. All analyses were conducted using the R software (version 4.3.1).

##### 2.5.3.3 Breathing Rate Changes

We examined whether voluntary movements significantly affected respiratory and heart rates. Respiratory intervals were measured during stimulus/action events and one interval before and after the movement, resulting in three intervals per movement. A two‐way ANOVA (condition × timing) was conducted for these intervals.

##### 2.5.3.4 Trial‐By‐Trial Respiratory Phase Coupling Between Stimulus Presentation and Voluntary Action (Libet Clock Task)

To clarify whether the respiratory phases temporally couple stimulus presentation and voluntary action initiation within trials, we calculated the circular correlations between the respiratory phases at dot onset (stimulus) and key press or release (action). Additionally, we examined the correlations between the respiratory phase at action timing in one trial and the respiratory phase at dot onset in the subsequent trial to assess potential trial‐by‐trial respiratory influences. To control for the possible confounding effects of inter‐event intervals, each phase angle was first decomposed into sine and cosine components. The linear effect of the interval was removed from each component via regression; the residual components were then recombined into a phase angle using the two‐argument arctangent function, and correlations were computed using these residual phases. The resulting correlation coefficients for each participant were converted to Fisher's *z*‐values, averaged across key‐press and key‐release conditions, and one‐sample *t*‐tests were performed to determine whether the group‐level mean *z*‐values significantly exceeded 0.

##### 2.5.3.5 Individual Difference Analysis for Respiration

To characterize individual variability in the respiratory synchronization during spontaneous voluntary movements, we examined the physiological factors influencing the respiratory phases immediately preceding voluntary movements. Two indices were computed for each participant and condition: phase difference (event phase minus baseline phase) and phase‐locking strength (event phase variability minus baseline phase variability; multiplied by −1 so that larger values indicate stronger phase‐locking). We first assessed within‐participant consistency by correlating these indices across voluntary action types and stimulus presentation timings. We then explored whether individual physiological characteristics– including age, mean breathing interval, breathing variability (RMSSD), R–R interval, and heart rate variability (RMSSD) – were associated with phase‐locking strength. To complement these correlation analyses, we additionally fitted participant‐level linear mixed‐effects models predicting phase‐locking strength from condition, age, gender, and the respiratory and cardiac variability indices listed above (see Supplementary Mixed‐effects Analyses).

## 3 Results

### 3.1 Libet Clock Task

#### 3.1.1 Basic Measurements

The average inhalation, exhalation, and total breathing cycle durations were 1.781 s (standard deviation [SD] = 0.422), 2.173 s (SD = 0.603), and 3.969 s (SD = 1.007), respectively. The correlation between the respiration belt and thermal airflow signals after alignment was 0.780 (SD = 0.125), with a thermal airflow delay of 382.5 ms (SD = 160.5). Approximately 5.5% (SD = 2.9) of the respiratory cycles were excluded as outliers. Regarding the ECG, the average R‐R interval was 0.800 s (SD = 0.100), with 2.2% (SD = 2.5) of R‐R intervals excluded as outliers. The ECG data from one participant were excluded because of measurement issues.

Approximately 7.7% of the trials were excluded based on predefined behavioral criteria. The final mean numbers of analyzable trials per participant were as follows: in the key‐press condition, 37.4 (SD = 2.5) for dot presentation, and 37.7 (SD = 2.1) for key press; in the key‐release condition, 36.1 (SD = 3.8) for dot presentation and 37.0 (SD = 2.8) for key release. Trial counts differed slightly across event types because, in addition to behavioral exclusions, events falling within excluded respiratory/cardiac cycles (after physiological outlier handling) were omitted from phase‐based analyses. The mean action intervals were 4.332 s (SD = 1.753) in the key‐press condition and 4.033 s (SD = 1.619) in the key‐release condition (Figure S4). The mean reaction time from dot presentation to key press under the key‐release condition was 0.414 s (SD = 0.105).

#### 3.1.2 Breathing Synchronization

##### 3.1.2.1 Circular Analysis

**FIGURE 3:**
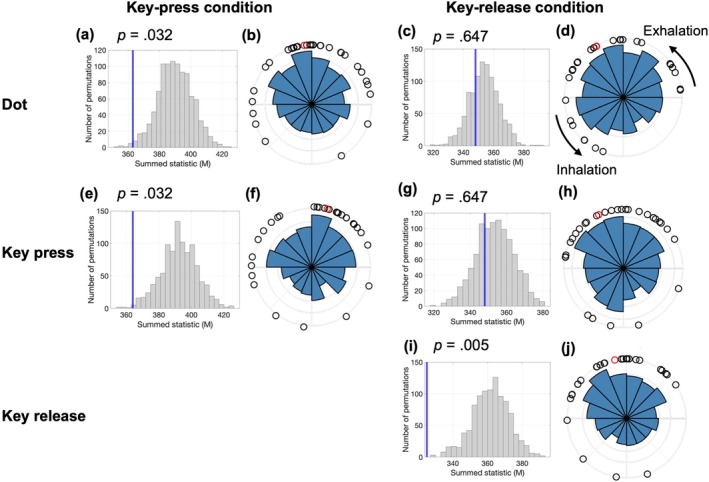
Respiratory synchronization and phase distributions during stimulus presentation and voluntary actions in the Libet clock task. Each horizontal row corresponds to a specific event type and experimental condition: (a, b) dot presentation in the key‐press condition; (c, d) dot presentation in the key‐release condition; (e, f) key press in the key‐press condition; (g, h) key press in the key‐release condition; and (i, j) key release in the key‐release condition. The left panels (a, c, e, g, i) show the surrogate data distributions of the summed statistics (*M*) as gray histograms, with the observed values indicated by vertical blue lines. Panels with significant effects show a leftward shift relative to the surrogate distribution (*p*‐values indicated in each panel). The right panels (b, d, f, h, j) display circular histograms illustrating the respiratory phase distributions of event timings across the trials. The black dots represent individual participants' mean respiratory phases, and the red dots indicate the overall group‐level mean phases.

We tested whether voluntary actions and stimulus presentation systematically aligned with the spontaneous breathing phases (Figure 3). In the key‐press condition, significant respiratory synchronization was observed at the dot presentation (*p* = 0.032) and key‐press (*p* = 0.032) timings. By contrast, in the key‐release condition, synchronization was significant only at the key‐release timing (*p* = 0.005) but not at the dot presentation or key‐press timings (*p*s > 0.60). These effects were robust to alternative preprocessing choices regarding respiratory pauses and outlier exclusion (Figures S8 and S9). All *p*‐values were corrected using the FDR method.

Moore's paired tests with FDR correction confirmed that in the key‐press condition, the mean respiratory phase at key‐press onset (*R* = 1.84, *p* < 0.001) and dot presentation (*R* = 1.69, *p* < 0.001) differed significantly from baseline, occurring earlier in the exhalation phase. In the key‐release condition, no significant differences were observed between the key‐release and baseline phases (*R* = 0.85, *p* = 0.17). Additionally, the mean respiratory phase did not differ significantly between key‐release and key‐press timings (*R* = 0.72, *p* = 0.23).

##### 3.1.2.2 State Analysis

**FIGURE 4:**
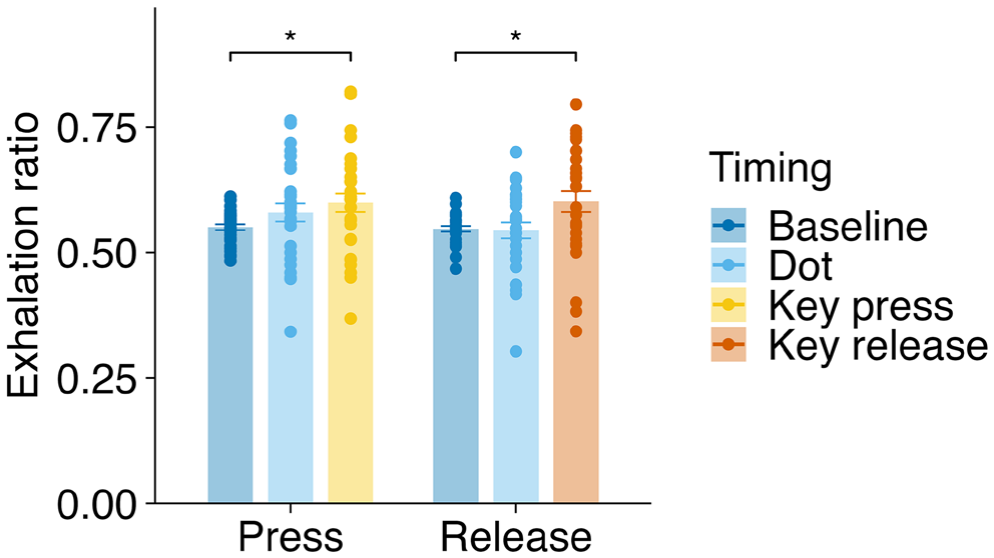
Exhalation ratios at different event timings in the Libet clock task. Mean exhalation ratios for baseline, dot presentation, and key‐press or key‐release timings in the key‐press and key‐release conditions. Individual participant data points are represented as dots, and the error bars indicate standard errors of the mean (SEM). Asterisks (*) denote significant differences (FDR‐corrected pairwise comparisons; *p < 0.05).

A two‐way ANOVA (condition × timing) revealed a significant main effect of timing (*F*(2, 54) = 7.96, *p* < 0.001, *ges* = 0.083), indicating differences in respiratory state across event timings (Figure 4). Neither the main effect of the condition (*F*(1, 27) = 1.77, *p* = 0.194, *ges* = 0.006) nor the interaction between the condition and timing (*F*(2, 54) = 1.39, *p* = 0.258, *ges* = 0.009) was significant. Follow‐up pairwise comparisons were FDR‐corrected. Compared with the baseline, the proportion of events occurring during exhalation significantly increased at key press in the key‐press condition (*t*(27) = 3.35, *p* = 0.005, *d* = 0.63) but not at dot presentation (*t*(27) = 1.55, *p* = 0.133, *d* = 0.29). In the key‐release condition, a similar significant increase was observed at key release (*t*(27) = 3.13, *p* = 0.008, *d* = 0.59), but no difference was found at dot presentation (*t*(27) = 0.38, *p* = 0.706, *d* = 0.07). Overall, these results indicated that voluntary key presses and releases occurred preferentially during exhalation relative to baseline.

##### 3.1.2.3 Breathing Rate Changes

**FIGURE 5:**
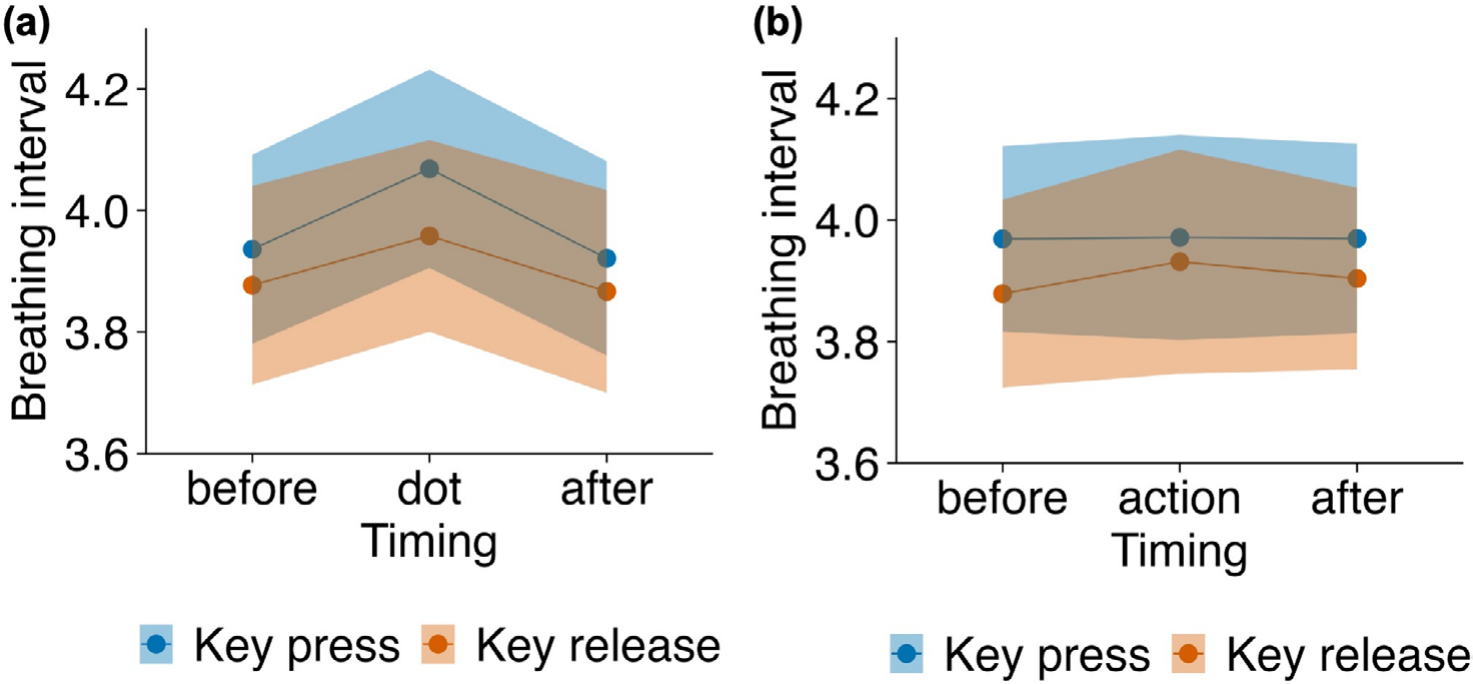
Breathing interval changes around stimulus presentations and voluntary actions in the Libet clock task. Mean breathing intervals (in seconds) are shown for the interval immediately before (Before1), during, and immediately after (After1) (a) dot presentations and (b) voluntary actions (key press/release). Shaded areas indicate standard errors of the mean.

Repeated‐measures ANOVAs were used to examine the breathing intervals around stimulus presentations and voluntary actions (Figure 5). Breathing intervals differed significantly across timing (main effect of timing: *F*(2, 54) = 8.36, *p* < 0.001, *ges* = 0.002), without significant effects of condition or interaction. Pairwise *t*‐tests confirmed that the intervals during dot presentation were significantly longer than those preceding stimulus onset (Before1) in the key‐press condition (*t*(27) = −3.23, *p* = 0.003, *d* = 0.61) but not in the key‐release condition (*t*(27) = −1.81, *p* = 0.082, *d* = 0.34). By contrast, the breathing intervals around voluntary actions showed no significant effects or interactions (*p*s > 0.375). These findings indicated that breathing was modulated around stimulus presentations but not voluntary actions.

##### 3.1.2.4 Trial‐By‐Trial Respiratory Phase Coupling Between Stimulus Presentation and Voluntary Action

**FIGURE 6:**
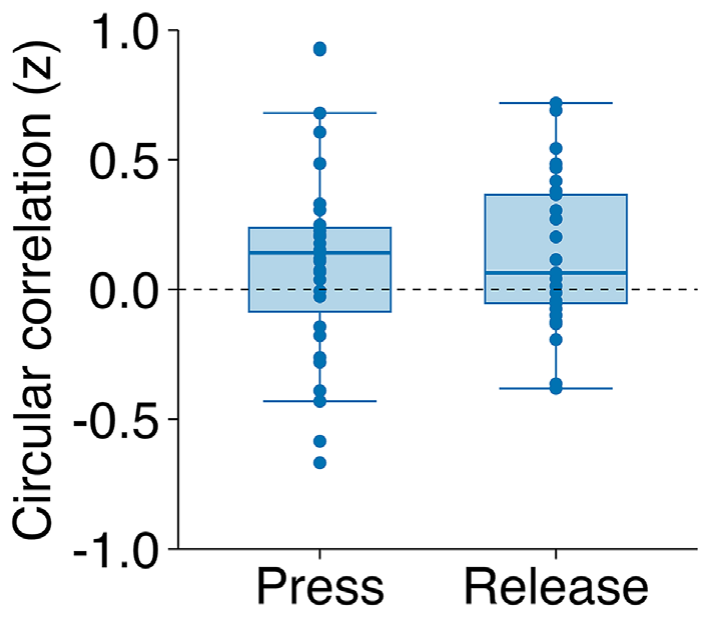
Circular correlations (Fisher's *z*‐transformed) between respiratory phases at dot presentation and subsequent voluntary actions. The conditions are shown on the horizontal axis: Key press and key release. Individual participant data points are indicated by dots. The boxes represent the IQR. The horizontal lines within the boxes indicate the medians, and the whiskers extend to 1.5 × IQR.

We examined the trial‐by‐trial relationships between respiratory phases at stimulus (dot) presentation and subsequent voluntary actions using circular correlation analyses. The results revealed a significant correlation between the respiratory phases at dot presentation and the subsequent voluntary action timings (*t*(27) = 2.62, *p* = 0.014, *d* = 0.495) (Figure 6). However, the respiratory phases during voluntary actions (key press or release) did not significantly predict the respiratory phases at dot presentation in the following trial (*t*(27) = 1.28, *p* = 0.213, *d* = 0.241). A trial‐wise GLMM with participant random intercepts and baseline exhalation offsets confirmed that only the respiratory state at dot onset predicted exhalation at action timing (Table S3).

##### 3.1.2.5 Individual Differences in Respiratory Synchronization

We examined individual differences in respiratory synchronization across the conditions. No significant correlation was observed between the key‐press and key‐release conditions in terms of the mean phase difference from baseline (*r* = 0.33, *p* = 0.091). However, the phase‐locking strength was significantly correlated between the two conditions (*r* = 0.38, *p* = 0.049). This suggests that participants consistently exhibited similar patterns of synchronization strength across different voluntary actions. Within the key‐press condition, individual differences in phase difference and phase‐locking strength were not significantly correlated between the dot presentation and key‐press timings (|*r*| < 0.12, *p* > 0.56). The exploratory analyses examining the correlations between individual differences in phase‐locking strength and physiological factors are presented in Table S1.

#### 3.1.3 Heart Synchronization

##### 3.1.3.1 Circular Analysis

**FIGURE 7:**
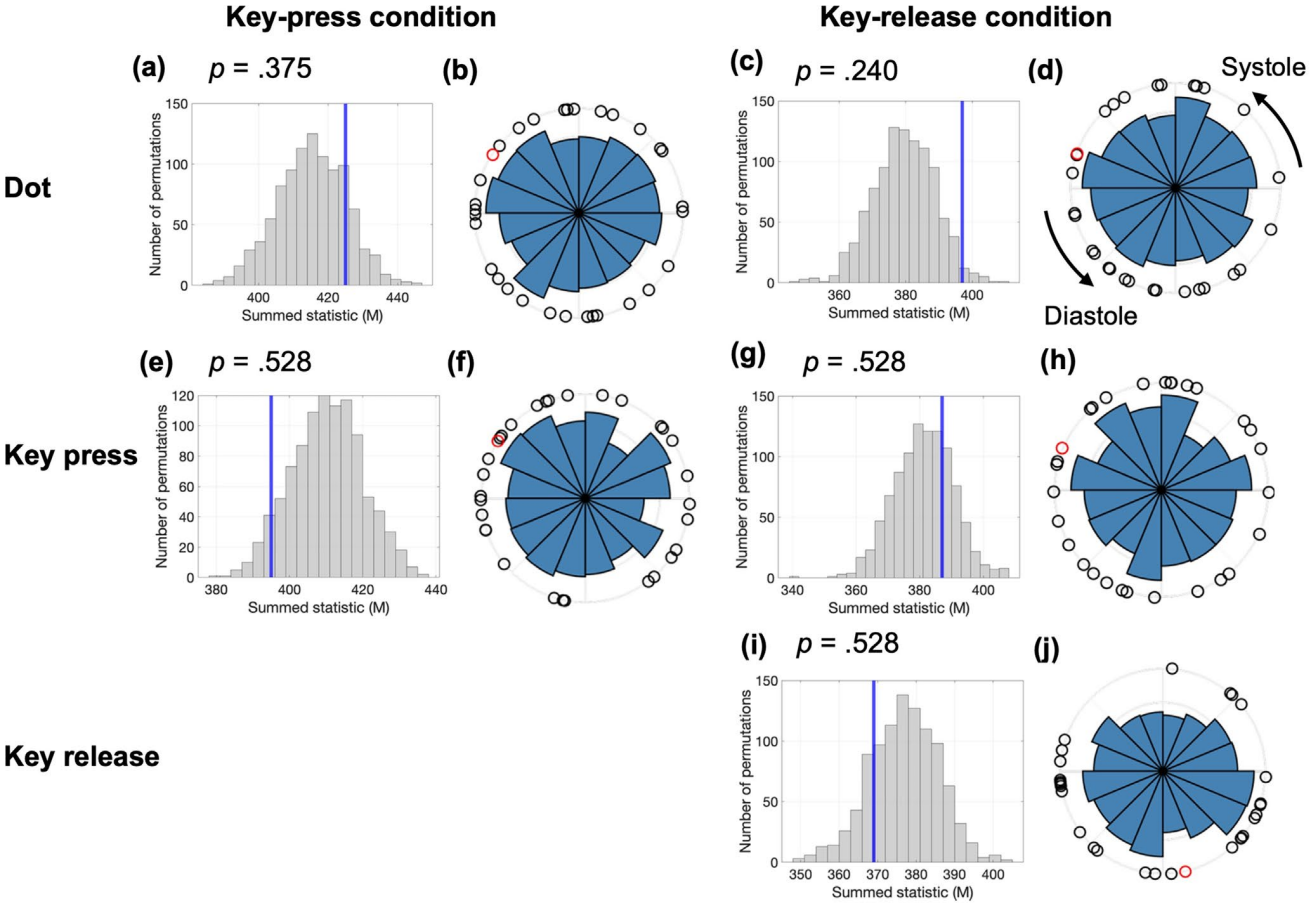
Cardiac synchronization and phase distributions during stimulus presentation and voluntary actions in the Libet clock task. Each horizontal row corresponds to a specific event type and experimental condition: (a, b) dot presentation in the key‐press condition; (c, d) dot presentation in the key‐release condition; (e, f) key press in the key‐press condition; (g, h) key press in the key‐release condition; and (i, j) key release in the key‐release condition. The left panels (a, c, e, g, i) show the surrogate data distributions of the summed statistics (*M*) as the gray histograms, with the observed values indicated by vertical blue lines. The right panels (b, d, f, h, j) display circular histograms illustrating the cardiac phase distributions of event timings across the trials. The black dots represent individual participants' mean cardiac phases, and the red dots indicate the overall group‐level mean phases.

We examined whether voluntary movements systematically aligned with the cardiac phases (Figure 7). The circular analyses (Hodges–Ajne tests with FDR correction) revealed no significant phase biases for timing or condition (*p* > 0.23).

A paired Moore's test with randomization indicated no significant difference between the cardiac phases at the key‐press timing in the key‐press condition and key‐release timing in the key‐release condition (*R* = 0.82, *p* = 0.146). These findings suggest that voluntary action timings were not significantly associated with specific cardiac phases.

##### 3.1.3.2 State Analysis

**FIGURE 8:**
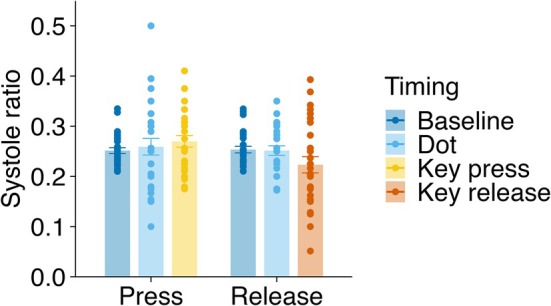
Cardiac systole ratio across conditions. Mean proportion of events occurring within 200–400 ms after the R‐peak (systole ratio) for baseline, dot presentation, and voluntary actions in the key‐press (Press) and key‐release (Release) conditions. Bars represent condition means and dots indicate individual participant data. Error bars represent SEM.

A two‐way ANOVA (condition × timing) on systole ratios (defined as the proportion of events occurring within 200–400 ms after the R‐peak) revealed a significant main effect of condition (*F*(1, 26) = 9.18, *p* = 0.005, *ges* = 0.028) but not timing (*F*(2, 52) = 0.64, *p* = 0.530, *ges* = 0.026) or interaction (*F*(2, 52) = 2.14, *p* = 0.129, *ges* = 0.026) (Figure 8). These results indicate that event timing was not robustly aligned with systole across conditions. Exploratory pairwise comparisons between each timing and its baseline showed no significant differences after FDR correction. The key‐release condition showed a numerical trend toward lower systole ratio at action timing (*t*(26) = 2.34, *p* = 0.027, *p*_.adj_ = 0.055, *d* = 0.45, BF_10_ = 2.15), but this did not survive correction for multiple comparisons. All other comparisons were non‐significant (*p* ≥ 0.145, all BF_10_ < 1). Taken together, these results provide no reliable evidence for cardiac‐phase modulation of voluntary action timing, although a weak trend in the key‐release condition cannot be entirely ruled out.

### 3.2 Elbow Flexion–Extension Task

#### 3.2.1 Basic Measurements

In the pull condition, the average inhalation, exhalation, and total breathing cycle durations were 1.852 s (SD = 0.621), 2.285 s (SD = 0.791), and 4.168 s (SD = 1.426), respectively. In the push condition, the averages were 1.786 s (SD = 0.607), 2.172 s (SD = 0.593), and 3.964 s (SD = 1.180), respectively. The correlation between the respiration belt and airflow signals after alignment was 0.709 (SD = 0.196), with an airflow delay of 455.1 ms (SD = 208.8). Approximately 5.2% (SD = 2.9) and 5.5% (SD = 3.8) of the respiratory cycles were excluded in the pull and push conditions, respectively.

Behaviorally, 6.3% of the trials were excluded. The final mean numbers of analyzable trials per participant were 39.0 (SD = 12.5) in the pull condition and 40.2 (SD = 11.6) in the push condition. The mean action intervals were similar for the pull (10.611 s, SD = 3.035) and push (10.586 s, SD = 3.053) actions (Figure S5).

#### 3.2.2 Breathing Synchronization

##### 3.2.2.1 Circular Analysis

**FIGURE 9:**
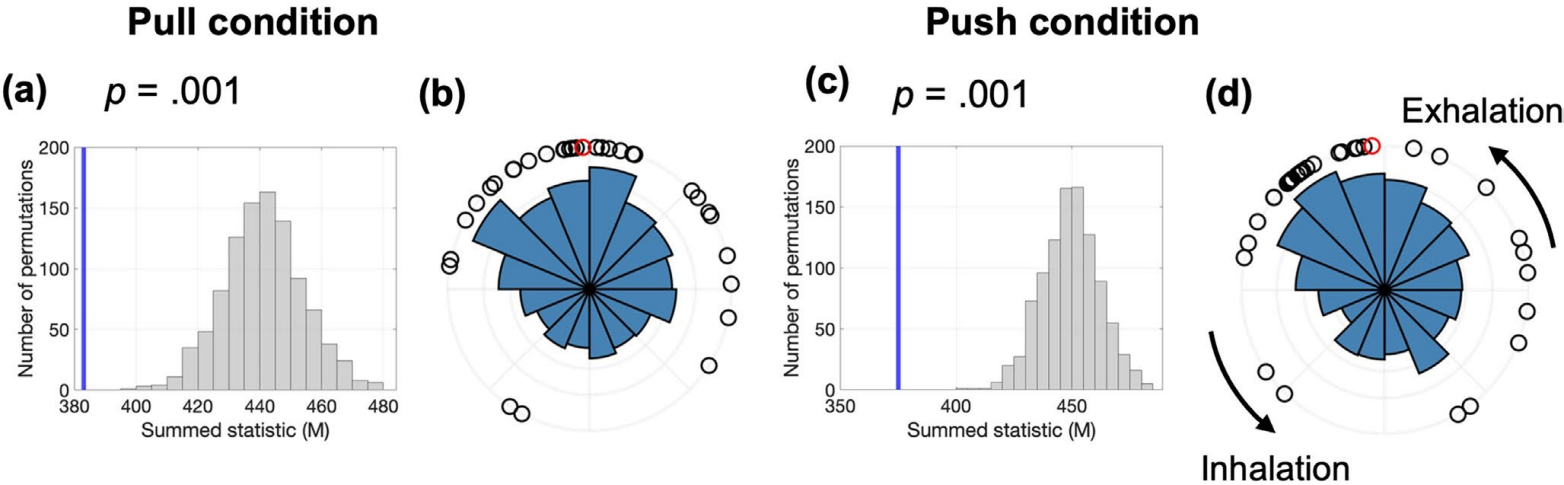
Respiratory synchronization and phase distributions during voluntary actions in the elbow flexion–extension task. Each horizontal row corresponds to a specific event type and experimental condition: (a, b) pull condition and (c, d) push condition. The left panels (a, c) show the surrogate data distributions of the summed statistics (*M*) as gray histograms, with the observed values indicated by vertical blue lines. The observed values shifted significantly to the left, indicating stronger synchronization than that expected by chance (associated *p*‐values are shown). The right panels (b, d) display circular histograms illustrating the respiratory phase distributions of event timings across trials. The black dots represent individual participants' mean respiratory phases, and the red dots indicate the overall group‐level mean phases.

We examined whether voluntary elbow movements were synchronized with the respiratory phases (Figure 9). The Hodges–Ajne test revealed significant respiratory phase biases at movement onset for the pull and push conditions (*p* = 0.001, FDR‐corrected). These effects were robust to alternative preprocessing choices regarding respiratory pauses and outlier exclusion (Figures S10 and S11).

Moore's paired test showed that the respiratory phase at push onset differed significantly from baseline (*R* = 1.138, *p* = 0.047). However, pull onset was not significantly different (*R* = 0.926, *p* = 0.086). No significant phase difference was observed directly between the pull and push movements (*R* = 0.595, *p* = 0.362).

##### 3.2.2.2 State Analysis

**FIGURE 10:**
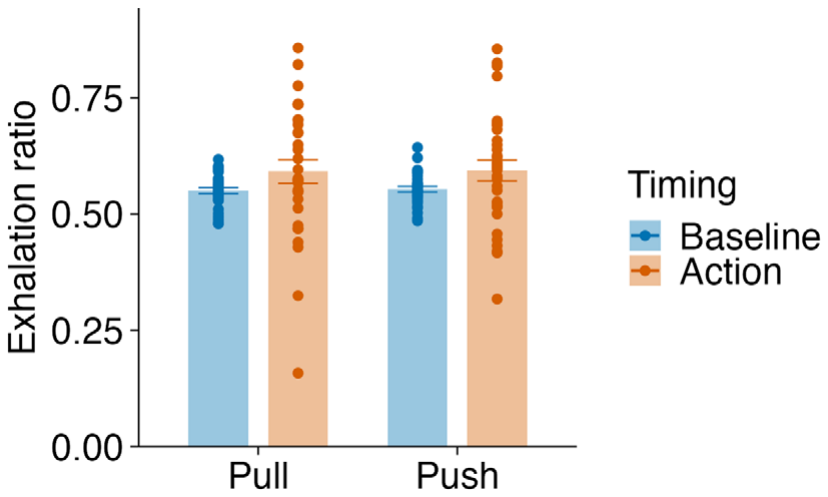
Exhalation ratios at different event timings in the elbow flexion–extension task. The mean exhalation ratios for baseline and pull or push timing. Individual participant data points are represented as dots, and the error bars indicate standard errors of the mean.

A two‐way ANOVA (condition × timing) revealed a significant main effect of timing (*F*(1, 31) = 5.54, *p* = 0.025, *ges* = 0.042) but no significant main effect of condition (*F*(1, 31) = 0.04, *p* = 0.849, *ges* < 0.001) or a significant interaction (*F*(1, 31) < 0.01, *p* = 0.975, *ges* < 0.001) (Figure 10). Pairwise comparisons showed that exhalation ratios at movement onset did not significantly differ from baseline for the pull (*t*(31) = 1.76, *p* = 0.089, *d* = 0.310) or push (*t*(31) = 1.95, *p* = 0.060, *d* = 0.345) conditions. An analogous GLMM for the elbow task did not identify any significant predictors of exhalation probability at movement onset (Table S4). These findings indicated a general trend toward increased exhalation at movement onset, accompanied by notable individual differences.

##### 3.2.2.3 Breathing Rate Changes

We used repeated‐measures ANOVAs to examine the breathing intervals surrounding voluntary movements (Figure S6). For the breathing intervals around voluntary movements, a two‐way repeated‐measures ANOVA revealed no significant main effect of timing (*F*(2, 62) = 0.32, *p* = 0.725, *ges* < 0.001) or condition (*F*(1, 31) = 3.86, *p* = 0.058, *ges* = 0.004) nor a significant interaction (*F*(2, 62) = 0.27, *p* = 0.764, *ges* < 0.001).

##### 3.2.2.4 Individual Differences

We analyzed the individual differences in respiratory synchronization between the pull and push conditions. The mean phase difference (*r* = 0.18, *p* = 0.323) and phase‐locking strength (*r* = 0.22, *p* = 0.244) were not significantly correlated across conditions. The exploratory analyses showed positive correlations between breathing variability (RMSSD) and phase‐locking strength in both the pull (*r* = 0.53, *p* = 0.002) and push (*r* = 0.43, *p* = 0.017) conditions, suggesting that participants with higher breathing variability exhibited stronger synchronization. Additional correlations are presented in Table S2. A participant‐level LMM similarly supported the main pattern, identifying breathing variability as the only meaningful predictor of phase‐locking strength while showing no additional robust associations (Table S5).

##### 3.2.2.5 Technical Considerations

Post‐experimental verification was conducted by repeating the measurements for six participants. Results indicated average measurement delays of 201.1 ms (SD = 93.1) in the pull condition and 218.7 ms (SD = 83.8) in the push condition. These delays reflect technical limitations associated with the threshold‐based detection method used to identify precise action onset timings.

## 4 Discussion

This study examined whether respiratory and cardiac signals are coupled with multiple voluntary actions and stimulus presentations. We found (i) voluntary actions synchronizing with exhalation across different effectors, (ii) dissociation and interaction between the stimulus and action‐locked coupling with breathing, and (iii) no robust evidence for systematic cardiac‐phase coupling of actions, with at most a weak and condition‐specific trend.

### 4.1 Exhalation Dominance Across Effectors

The results demonstrated an overall exhalation‐phase bias for the initiation of voluntary actions across effectors (finger and elbow) and movement direction (flexion and extension), with notable individual differences. Importantly, we found no respiration–action coupling in the triggered key press, consistent with Park et al. (2020). These findings replicate the exhalation bias previously reported for self‐initiated key presses (Park et al. 2020) and extend it by showing generalizability across distinct effectors and kinematic profiles. The observation that exhalation‐aligned timing is present for key releases and elbow movements, in addition to key presses, suggests that this alignment may reflect a common influence on action initiation rather than a property restricted to a single motor action.

Physiological and neuroscientific evidence supports the notion that voluntary actions are preferentially initiated during exhalation. Physiologically, inhalation is an active phase driven by the contraction of inspiratory pump muscles (e.g., diaphragm), involving considerable metabolic cost (del Negro et al. 2018). This phase generates a surge of afferent signals from pulmonary mechanoreceptors and the olfactory bulb, which modulate central neural processing (Brændholt et al. 2023; del Negro et al. 2018). By contrast, exhalation during resting breathing is largely passive and energetically inexpensive, relying on elastic recoil (del Negro et al. 2018). This passive state minimizes competition between respiratory and voluntary motor processes, potentially facilitating voluntary action initiation. From a neuroscientific perspective, studies have indicated that cortical states vary systematically with respiratory phases, reflecting the respiration‐driven entrainment of cortical oscillatory networks across multiple frequency bands (Brændholt et al. 2023; Tort et al. 2018, 2025). Specifically, recent evidence demonstrates that respiration modulates cortical excitability (Kluger et al. 2021, 2023). This respiratory modulation extends to motor preparation: RP associated with voluntary action preparation covaries systematically with respiratory phases (Park et al. 2020, 2022), and motor evoked potentials vary with the respiratory cycle (Engelen et al. 2025). Collectively, these findings suggest differentiated psychophysiological roles of inhalation and exhalation: exhalation provides a favorable temporal window for the initiation of voluntary actions.

The time course of motor preparation may interact with respiration–action coupling. Voluntary movements are preceded by preparatory modulation of corticospinal excitability, including transient suppression in the several hundred milliseconds before movement onset (Duque and Ivry 2009; Greenhouse et al. 2015). Speculatively, this preparatory interval may offer a temporal window in which respiratory phase can bias action timing when preparation time is available, whereas such phase‐dependent biases may be reduced under high temporal urgency when preparation is constrained (Greenhouse et al. 2015; Haith et al. 2016). This framework is consistent with the absence of respiration–action coupling in our triggered key press where preparation time was limited.

A purely biomechanical account, in which movements align with breathing phases optimized for energetic efficiency, cannot adequately explain the observed results. Early isometric studies demonstrated that finger flexor force is greater during forced exhalation than forced inhalation (Li and Laskin 2006); similarly, elbow extension torque also peaks during forced exhalation, whereas elbow flexion torque shows no clear respiratory phase dependency (Ikeda et al. 2009). Studies on finger‐tracking movements indicate differing respiratory influences on the precision of flexion and extension movements (Rassler 2000). If respiratory coupling is primarily driven by biomechanical optimization, distinct effectors should display distinct respiratory couplings; however, this pattern was not observed in our study. Instead, the consistent alignment of various voluntary actions with exhalation supports the notion that respiration functions as a temporal cue at the level of action intention, preceding effector‐specific motor planning and execution.

Notably, individual participant traits may influence respiration–action coupling. Despite the general trend toward exhalation alignment, considerable individual variability was observed. For instance, in the elbow flexion–extension task, some participants exhibited robust synchronization with inhalation rather than exhalation, consistent with recent findings that the optimal respiratory phase for motor output varies substantially across individuals (Engelen et al. 2025). Such variability may reflect individual differences in respiratory control networks or learned motor patterns. Additionally, we identified a positive correlation of the phase‐locking strength between the key‐press and key‐release conditions in the Libet clock task. This finding suggests that participants may possess individual‐specific tendencies, potentially reflecting broader psychophysiological traits such as interoceptive sensitivity. Indeed, individual respiratory patterns exhibit high temporal stability and predict various physiological and cognitive characteristics (Soroka et al. 2025), suggesting that respiration–action coupling may similarly reflect stable individual traits. Physiological factors were also correlated with phase‐locking strength. Breathing variability was positively correlated with synchronization strength in the elbow flexion–extension task, suggesting that higher breathing flexibility may broaden the temporal window for aligning actions with preferred respiratory phases. Clarifying these individual‐specific relationships through systematic assessment of interoceptive traits and respiratory characteristics is a promising direction for future research.

### 4.2 Dissociable Respiratory Coupling With Stimulus Presentation and Voluntary Action

When stimulus‐locked coupling was observed, stimulus onsets and subsequent voluntary actions tended to occur within a similar mid‐ to late‐exhalation window, suggesting that respiration may provide a shared temporal reference within each trial. The circular correlation analyses confirmed significant trial‐by‐trial coupling between the respiratory phase at stimulus onset and subsequent voluntary action timing; however, no carry‐over was observed between voluntary actions and subsequent stimulus presentations. These findings are consistent with the possibility that the respiratory phase at stimulus onset establishes a trial‐specific temporal reference, a respiratory‐based temporal framework, relative to which voluntary action timing may be organized.

Although stimulus‐ and action‐locked couplings share the same respiratory phase window, the underlying control processes appear to be distinct. The stimulus‐locked effect was accompanied by prolonged breathing intervals around stimulus onset, consistent with an adjustment of breathing to the trial structure. By contrast, breathing intervals were unchanged around voluntary actions, implying that action timing was biased by ongoing respiratory rhythms rather than actively altering the respiratory cycle. Notably, such stimulus‐locked adjustment of breathing was weaker in the key‐release condition, possibly because immediate motor responses constrained the opportunity for exhalation prolongation. Moreover, individual differences in stimulus‐ and action‐locked synchronization were uncorrelated, consistent with distinct underlying mechanisms for these processes.

Additionally, the direction of stimulus synchronization depends on the task characteristics. Johannknecht and Kayser (2022) reported stimulus alignment with inhalation in memory tasks, which they interpreted as reflecting active respiratory adjustments to optimize cognitive performance (Perl et al. 2019; Zelano et al. 2016). By contrast, stimulus synchronization in our Libet clock task, which lacked task‐relevant stimulus information, predominantly occurred during exhalation, likely representing passive entrainment facilitated by a flexible exhalation duration. Thus, even randomized inter‐trial intervals ranging from 4 to 8 s, commonly assumed to prevent rhythmic entrainment, can unintentionally overlap with the participants' breathing cycles. Importantly, respiration–action coupling was observed in both the Libet clock task (with stimuli) and the elbow flexion–extension task (without external stimuli), suggesting that intrinsic respiration–action coupling may reflect properties of action initiation, with stimulus‐locked modulation providing an additional influence in stimulus‐structured tasks.

### 4.3 Cardiac Phase Coupling on Voluntary Action

Evidence for the cardiac synchronization of voluntary action initiation remains inconsistent, likely owing to methodological and task‐based differences. While Park et al. (2020), using the Libet clock task, found no systematic cardiac‐phase bias during self‐paced actions, Mussini et al. (2024) reported that participants tended to refrain from initiating voluntary responses during systole, instead favoring diastole. This finding is consistent with the baroreceptor‐mediated inhibitory theories (Makowski et al. 2020; Rae et al. 2018). Other studies have reported different patterns, such as increased action frequency during systole (Kunzendorf et al. 2019) or a tendency to act away from the R‐peak (Palser et al. 2021). These discrepancies may reflect differences in whether actions were purely self‐initiated, whether subsequent stimuli shaped action timing, or whether the outcome of interest was initiation versus offset.

Employing the Libet clock task, we observed no statistically significant cardiac‐phase coupling across conditions. State‐based analyses revealed a numerical trend for key releases to occur less often during systole than at baseline, although this effect did not survive correction for multiple comparisons and Bayes factors provided only anecdotal evidence. This weak trend is directionally consistent with Mussini et al. (2024), providing suggestive but inconclusive evidence for a potential inhibitory influence of systole on voluntary action initiation. This effect was observed specifically in the key‐release condition, in which participants maintained continuous key contact before responding. This sustained engagement may have heightened sensitivity to cardiac signals, consistent with findings that task engagement modulates cardiac‐phase effects (Mussini et al. 2024; Yang et al. 2023). Overall, any cardiac‐phase modulation appears considerably smaller and less reliable than the respiratory effects observed here. Future studies with higher statistical power and designs optimized for cardiac‐phase effects will be needed to clarify these influences.

### 4.4 Limitations and Conclusion

First, the timing of measurement in the elbow flexion–extension task made it difficult to infer precise action onset timings because we relied on threshold‐based detection of the participants' movements. Post‐experimental verification in a subset of six participants indicated delays of approximately 200 ms between actual movement onset and our threshold‐based detection. As the respiratory cycles range from 3000 to 5000 ms, these delays are unlikely to substantially affect respiratory‐related conclusions. However, owing to the higher temporal sensitivity of the cardiac phases, we could not reliably analyze cardiac synchronization data from the elbow flexion–extension task. Second, we did not assess individual traits such as sports experience and interoceptive sensitivity. These variables may contribute to between‐participant variability in respiratory phase‐locking strength; therefore, the lack of these measures limits our ability to identify stable predictors of individual differences. Moreover, investigating subjective experiences such as feelings of action initiation or a sense of agency across different breathing phases could further elucidate individual differences. Third, we did not collect systematic post‐task subjective reports regarding voluntary breath control, awareness of respiration–action coupling, or task‐related strategies. Although participants were instructed to maintain natural nasal breathing and informal debriefings suggested little or no awareness of coupling, we cannot definitively rule out the possibility that subtle awareness or unreported strategies contributed to the phase‐locking in the absence of standardized introspective measures. Finally, we did not examine other types of voluntary actions that require varying degrees of power or precision. Given that different motor demands may yield divergent synchronization patterns, future studies should systematically explore these variations.

In conclusion, the current study advances our understanding of respiratory and cardiac influences on voluntary action by demonstrating (i) general exhalation–action coupling across various effectors and kinematics, (ii) the dissociation and integration of stimulus‐ and action‐related phase couplings, and (iii) no robust evidence for systematic cardiac‐phase coupling, with at most a weak, condition‐specific trend.

## Author Contributions

**Hiroshi Shibata:** conceptualization, methodology, data curation, formal analysis, investigation, writing – original draft, writing – review and editing, funding acquisition, visualization, validation. **Hideki Ohira:** funding acquisition, conceptualization, methodology, supervision, writing – review and editing, resources, validation.

## Funding

This study was supported by the JSPS KAKENHI (grant number 23KJ1078).

## Conflicts of Interest

The authors declare no conflicts of interest.

## Supporting information


**Figure S1:** Alignment of respiration signals from thermal airflow and respiration belt.
**Figure S2:** Determination of instantaneous respiratory phases using the Hilbert transform.
**Figure S3:** Determination of instantaneous cardiac phases from ECG data.
**Figure S4:** Waiting times before voluntary actions in the Libet clock Task.
**Table S1:** Correlation between individual difference of phase‐locking strength and physiological factors in the Libet clock task.
**Figure S5:** Waiting times before voluntary actions in the elbow flexion‐extension task.
**Figure S6:** Breathing interval changes around voluntary actions in the elbow flexion‐extension task.
**Table S2:** Correlation between individual difference of phase‐locking strength and physiological factors in the elbow flexion‐extension Task.
**Table S3:** Fixed effects from the GLMM predicting the likelihood of actions occurring during exhalation in the Libet clock task.
**Table S4:** Fixed effects from the GLMM predicting the likelihood of actions occurring during exhalation in the elbow flexion–extension task.
**Table S5:** Fixed effects from the linear mixed‐effects model predicting phase‐locking strength.
**Figure S8:** Sensitivity analysis of respiratory synchronization in the Libet task after excluding respiratory pauses.
**Figure S9:** Sensitivity analysis of respiratory synchronization in the Libet task without exclusion of respiratory‐cycle outliers.
**Figure S10:** Sensitivity analysis of respiratory synchronization in the elbow flexion–extension task after excluding long respiratory pauses.
**Figure S11:** Sensitivity analysis of respiratory synchronization in the elbow flexion–extension task without exclusion of respiratory‐cycle outliers.

## Data Availability

The data that support the findings of this study are not publicly available due to ethical restrictions. The analysis code is openly available at the GitHub repository listed below (https://github.com/baniki/phase‐coupling‐motor‐tasks).
