# Supplementary material for: Respiratory and Cardiac Phase Coupling With Voluntary Actions Across Motor Tasks
Source: Psychophysiology. 2026 Feb 18;63(2):e70264. doi: 10.1111/psyp.70264 (PMC12914617; doi:10.1111/psyp.70264)
Supplement: Supplementary file 1 — Figure S1: Alignment of respiration signals from thermal airflow and respiration belt. Figure S2: Determination of instantaneous respiratory phases using the Hilbert transform. Figure S3: Determination of instantaneous cardiac phases from ECG data. Figure S4: Waiting times before voluntary actions in the Libet clock Task. Table S1: Correlation between individual difference of phase‐locking strength and physiological factors in the Libet clock task. Figure S5: Waiting times before voluntary actions in the elbow flexion‐extension task. Figure S6: Breathing interval changes around voluntary actions in the elbow flexion‐extension task. Table S2: Correlation between individual difference of phase‐locking strength and physiological factors in the elbow flexion‐extension Task. Table S3: Fixed effects from the GLMM predicting the likelihood of actions occurring during exhalation in the Libet clock task. Table S4: Fixed effects from the GLMM predicting the likelihood of actions occurring during exhalation in the elbow flexion–extension task. Table S5: Fixed effects from the linear mixed‐effects model predicting phase‐locking strength. Figure S8: Sensitivity analysis of respiratory synchronization in the Libet task after excluding respiratory pauses. Figure S9: Sensitivity analysis of respiratory synchronization in the Libet task without exclusion of respiratory‐cycle outliers. Figure S10: Sensitivity analysis of respiratory synchronization in the elbow flexion–extension task after excluding long respiratory pauses. Figure S11: Sensitivity analysis of respiratory synchronization in the elbow flexion–extension task without exclusion of respiratory‐cycle outliers. [file PSYP-63-e70264-s001.docx]

**Supplementary Materials**

**Analyses
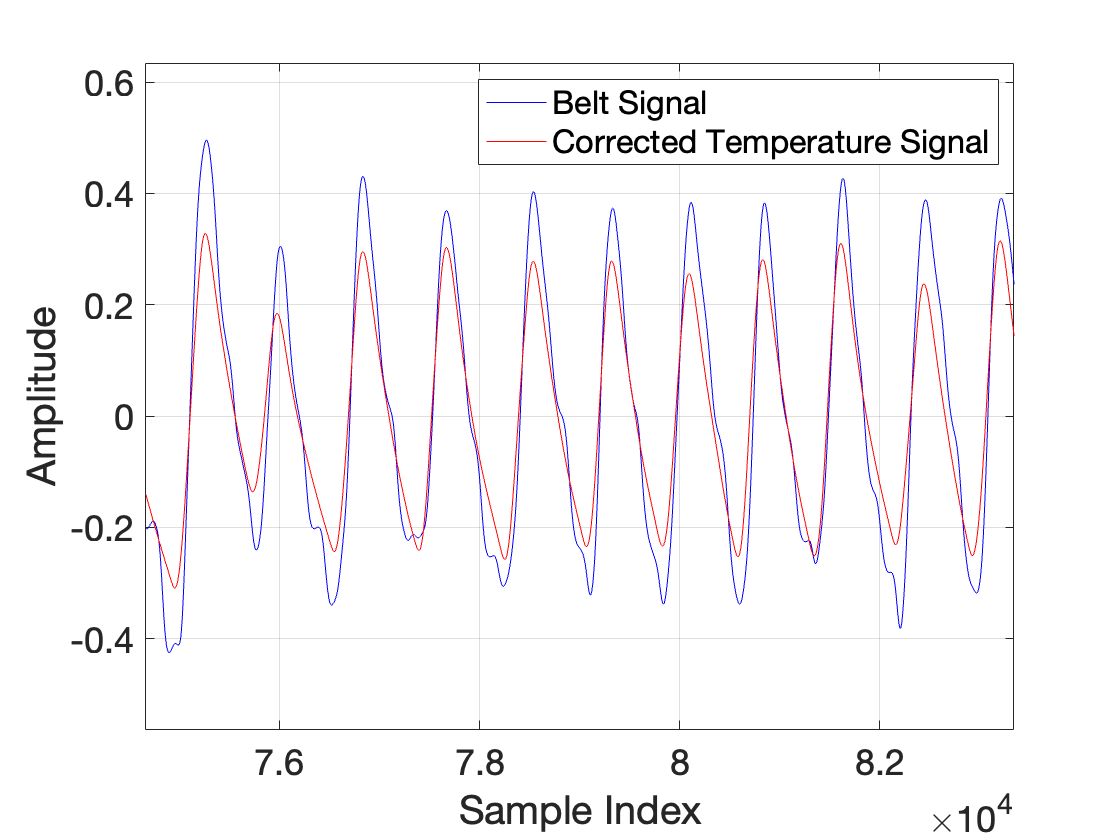
**

**Supplementary Figure 1. Alignment of respiration signals from thermal airflow and respiration belt.**

Representative example illustrating the correction procedure for thermal airflow signals. Due to temporal delay in thermal measurements, airflow signals were inverted in phase and temporally shifted based on cross-correlation analysis with the respiration belt signal. The figure shows raw respiration belt data (blue) and corrected thermal airflow signal (red), confirming optimal alignment between the two methods.

**Supplementary Figure 2. Determination of instantaneous respiratory phases using the Hilbert transform.**

(a) Example of band-pass filtered (0.1–3 Hz) respiratory signal (blue) and corresponding instantaneous respiratory phase (green), derived using the Hilbert transform. (b) Identification of inhalation and exhalation phases: peaks and troughs detected from the filtered respiratory signal define inhalation phases (red line) and exhalation phases (blue line). Green circles illustrate examples of event timings (e.g., voluntary actions) within the respiratory cycle.

**
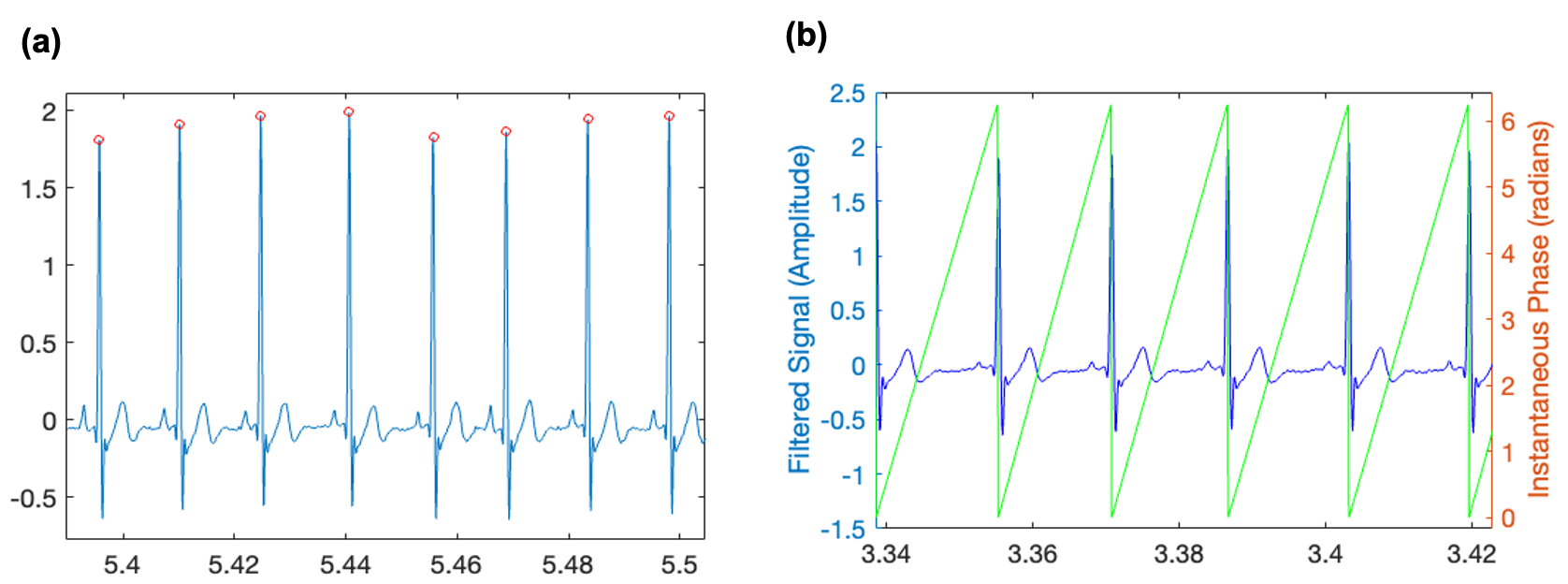
**

**Supplementary Figure 3. Determination of instantaneous cardiac phases from ECG data.**

(a) Example of ECG signal after band-pass filtering (0.5–40 Hz), with detected R-peaks indicated by red circles. (b) Corresponding instantaneous cardiac phase (green line), derived from detected R-peaks, used for synchronization analyses.

**Results**

**Libet clock task**

**Supplementary Figure 4. Waiting times before voluntary actions in the Libet clock Task.**

The histogram of waiting times (in seconds) prior to performing voluntary actions are shown separately for the (a) key-press and (b) key-release conditions.

**Supplementary Table 1. Correlation between individual difference of phase-locking strength and physiological factors in the Libet clock task**

|  | Stimulus coupling in key-press condition | Key-press coupling in key-press condition | Key-release coupling in key-release condition |
| --- | --- | --- | --- |
| Age | 0.21 | -0.14 | -0.18 |
| Breathing Interval | -0.16 | -0.14 | -0.24 |
| Breathing Variability (RMSSD) | 0.20 | 0.17 | -0.20 |
| R-R interval | -0.38* | -0.07 | 0.39* |
| Heart Rate Variability (RMSSD) | 0.18 | 0.04 | 0.09 |

**p* < .05

**Elbow flexion-extension task**

**Supplementary Figure 5. Waiting times before voluntary actions in the elbow flexion-extension task.**

The histogram of waiting times (in seconds) prior to performing voluntary actions are shown separately for the (a) pull and (b) push conditions.

**
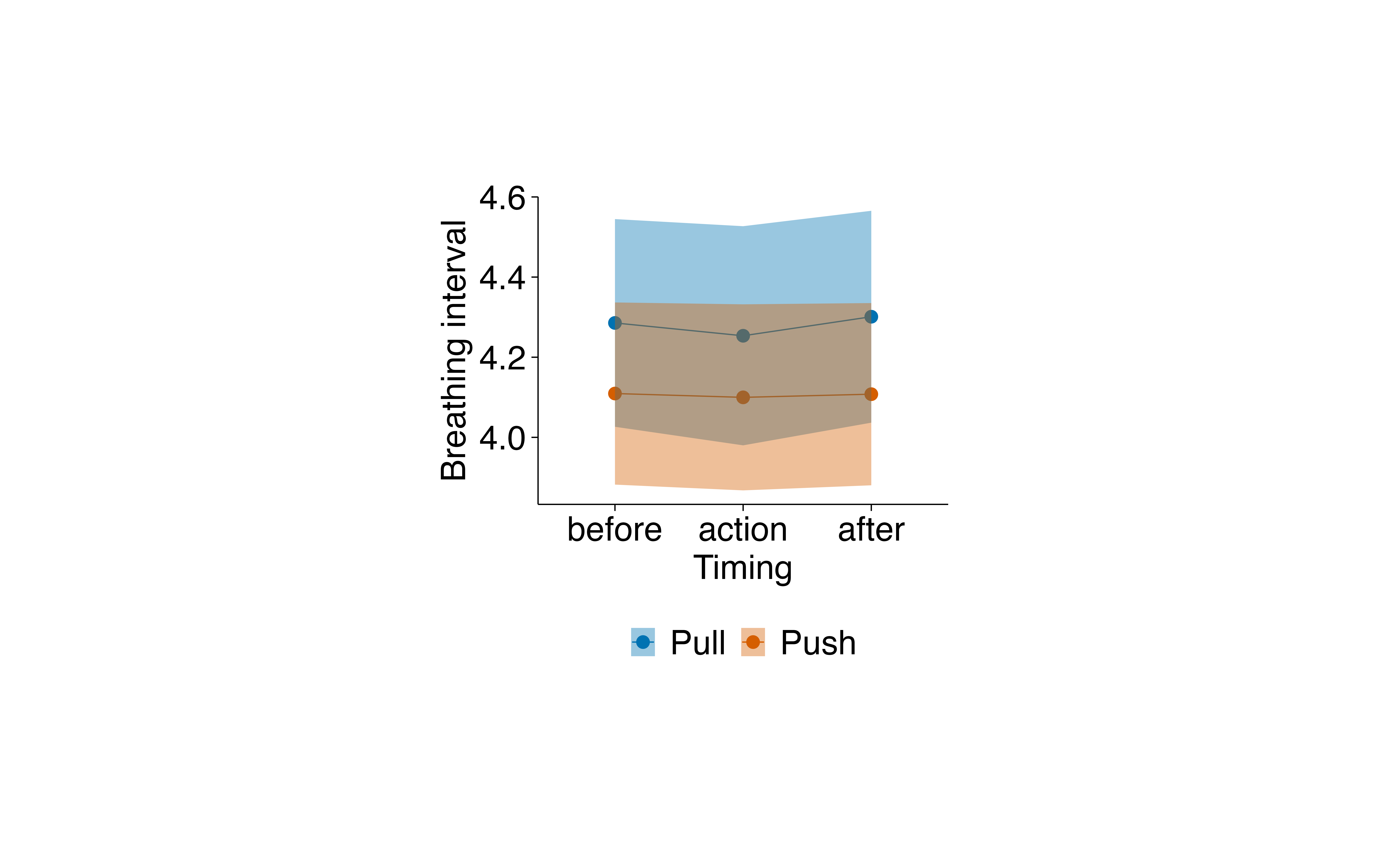
**

**Supplementary Figure 6. Breathing interval changes around voluntary actions in the elbow flexion-extension task.**

Mean breathing intervals (in seconds) are shown for the interval immediately before (Before1), during, and immediately after (After1) voluntary actions (pull/push). Shaded areas indicate standard errors of the mean.

**Supplementary Table 2. Correlation between individual difference of phase-locking strength and physiological factors in the elbow flexion-extension Task**

|  | Pull condition | Push condition |
| --- | --- | --- |
| Age | -0.14 | -0.18 |
| Breathing Interval | 0.39 | 0.28 |
| Breathing Variability (RMSSD) | 0.53* | 0.43* |
| R-R interval | 0.06 | 0.11 |
| Heart Rate Variability (RMSSD) | -0.11 | -0.08 |

**p* < .05

**Supplementary Mixed-effects analyses**

**Trial-wise GLMMs for respiratory coupling**

**Libet clock task**

To complement the circular and state-based analyses, we fitted a trial-wise generalized linear mixed-effects model (GLMM) to predict whether actions occurred during exhalation (0 = inhalation, 1 = exhalation). Fixed effects included condition (key-press vs key-release), respiratory state at dot presentation (exhalation vs inhalation), cardiac state at action timing, and the action interval. Random intercepts were included for participants, and each participant’s logit-transformed baseline exhalation ratio was entered as an offset:

$$ActionRespState\boldsymbol{\sim}Condition\boldsymbol{+}DotRespState\boldsymbol{+}CardiacState\boldsymbol{+}ActionInterval \boldsymbol{+ (1|}Participant\boldsymbol{) + offset(}BaselineLogit\boldsymbol{)}$$

As shown in Supplementary Table 3, the respiratory state during dot presentation significantly predicted the probability of exhalation at action timing, whereas condition, cardiac state, and action interval did not show reliable additional contributions. This finding is consistent with both the state-based analyses and the trial-by-trial respiratory phase–coupling analysis, which similarly indicated that the respiratory phase at dot onset was related to subsequent action timing.

**Supplementary Table 3. Fixed effects from the GLMM predicting the likelihood of actions occurring during exhalation in the Libet clock task**

| Predictor | Estimate | SE | z | p |
| --- | --- | --- | --- | --- |
| (Intercept) | 0.225 | 0.179 | 2.490 | .013 |
| Condition (Release) | 0.020 | 0.094 | 0.216 | .829 |
| DotRespState  (Inhalation) | -0.238 | 0.093 | -2.559 | .011 |
| CardiacState  (Not systole) | 0.086 | 0.106 | 0.813 | .416 |
| ActionInterval | -0.040 | 0.031 | -1.287 | .198 |

**Elbow flexion-extension task**

For the elbow flexion–extension task, we fitted an analogous GLMM with movement condition (pull vs push) and action interval as fixed effects:

$$ActionRespState\boldsymbol{\sim}Condition\boldsymbol{+}ActionInterval\boldsymbol{+ (1|}Participant\boldsymbol{)+ offset(}BaselineLogit\boldsymbol{)}$$

As shown in Supplementary Table 4, neither movement condition nor action interval significantly modulated the probability of exhalation at movement onset.

**Supplementary Table 4. Fixed effects from the GLMM predicting the likelihood of actions occurring during exhalation in the elbow flexion–extension task**

| Predictor | Estimate | SE | z | p |
| --- | --- | --- | --- | --- |
| (Intercept) | 0.338 | 0.196 | 1.725 | .085 |
| Condition (Push) | -0.042 | 0.082 | -0.508 | .612 |
| ActionInterval | -0.011 | 0.016 | -0.663 | .508 |

**Individual-differences LMM**

To investigate whether individual differences in respiratory phase-locking strength were related to demographic or physiological variables, we fitted a linear mixed-effects model:

$$\boldsymbol{PhaseLockingStrength \sim Condition + BreathVar+ HRV + Gender + Age+ (1|}Participant\boldsymbol{)}$$

As shown in Supplementary Table 5, none of the predictors showed robust associations with the phase-locking strength, except for breathing variability (Estimate = 0.100, *p* = .002), consistent with the pairwise correlation results presented in Supplementary Tables 1 and 2.

**Supplementary Table 5. Fixed effects from the linear mixed-effects model predicting phase-locking strength**

| Predictor | Estimate | SE | df | t | p |
| --- | --- | --- | --- | --- | --- |
| (Intercept) | 0.014 | 0.122 | 30.344 | 0.118 | .907 |
| Condition (Release) | 0.022 | 0.032 | 88.815 | 0.679 | .499 |
| Condition (Pull) | 0.039 | 0.031 | 88.271 | 1.240 | .218 |
| Condition (Push) | 0.033 | 0.032 | 87.660 | 1.049 | .297 |
| BreathVar | 0.100 | 0.032 | 105.209 | 3.144 | .002 |
| HRV | −0.282 | 0.448 | 47.469 | −0.631 | .531 |
| Gender (Male) | 0.033 | 0.029 | 27.822 | 1.115 | .275 |
| Age | 0.002 | 0.006 | 31.205 | 0.362 | .720 |

**Sensitivity Analyses**

**Supplementary Figure 8. Sensitivity analysis of respiratory synchronization in the Libet task after excluding respiratory pauses.**

Each horizontal row corresponds to a specific event type and experimental condition: (a, b) dot presentation in the key-press condition; (c, d) dot presentation in the key-release condition; (e, f) key press in the key-press condition; (g, h) key press in the key-release condition; and (i, j) key release in the key-release condition. In this analysis, respiratory pauses were defined as periods of near-zero slope of the airflow lasting ≥ 500 ms and all events occurring during such pauses were excluded (4.3 % of the data). The left panels (a, c, e, g, i) show the surrogate distributions of the summed statistic M (gray histograms), with the observed values indicated by vertical blue lines. The right panels (b, d, f, h, j) display circular histograms of the respiratory phase at event timing; black dots indicate individual participants’ mean phases and red dots the group-level mean phase. The same pattern of significant synchronization as in the main analysis is evident: dot presentation and key press in the key-press condition, and key release in the key-release condition.

**Supplementary Figure 9. Sensitivity analysis of respiratory synchronization in the Libet task without exclusion of respiratory-cycle outliers.**

Same layout as Supplementary Figure 8. Here, no exclusion based on respiratory cycle duration (total, inhalation, or exhalation) was applied; all detected cycles contributed to the phase estimates. The permutation distributions of M (left panels) and circular phase histograms (right panels) closely match those of the main analysis, with significant phase clustering again confined to dot presentation and key press in the key-press condition and key release in the key-release condition.

**Supplementary Figure 10. Sensitivity analysis of respiratory synchronization in the elbow flexion–extension task after excluding long respiratory pauses.**

Panels (a, b) show the pull condition and panels (c, d) the push condition. Respiratory pauses (near-zero slope of the airflow ≥ 500 ms) were removed (4.1 % and 3.6 % in the pull and push conditions, respectively), and events occurring during pauses were excluded from the analysis. As in Supplementary Figures 8 and 9, the left panels depict the surrogate distributions of the summed statistic M with observed values marked by blue vertical lines, and the right panels show circular histograms of respiratory phase at movement onset, with black dots for individual means and red dots for the group mean. Both pull and push movements remain significantly phase-locked to the respiratory cycle, replicating the main analysis.

**Supplementary Figure 11. Sensitivity analysis of respiratory synchronization in the elbow flexion–extension task without exclusion of respiratory-cycle outliers.**

Same format as Supplementary Figure 10, but including all respiratory cycles irrespective of duration. The permutation distributions of M and the circular phase histograms again reveal significant phase clustering for both pull and push movements, indicating that the conclusions for the elbow task are robust to the treatment of respiratory outliers.
